# Tele-pharmacy Anticoagulation Clinic During COVID-19 Pandemic: Patient Outcomes

**DOI:** 10.3389/fphar.2021.652482

**Published:** 2021-09-09

**Authors:** Maha Al Ammari, Khalefa AlThiab, Manal AlJohani, Khizra Sultana, Nada Maklhafi, Hayel AlOnazi, Aswaq Maringa

**Affiliations:** ^1^ Department of Pharmacy Service, King Abdul Aziz Medical City (KAMC), Riyadh, Saudi Arabia; ^2^ Ministry Of National Guard Health Affair(MNGHA), Riyadh, Saudi Arabia; ^3^ King Abdullah International Medical Research Center (KAIMRC), Riyadh, Saudi Arabia; ^4^ King Saud bin Abdulaziz University for Health Sciences (KSAU-HS), Riyadh, Saudi Arabia

**Keywords:** tele-pharmacy, anticoagulation, virtual clinic, international normalized ratio (INR), time in therapeutic range (TTR), patient outcomes, COVID-19, patient satisfaction

## Abstract

**Introduction:** It is well-established that clinical pharmacist-managed anticoagulation services achieve superior anticoagulation control, with a positive impact. At King Abdulaziz Medical City (KAMC), Riyadh, Saudi Arabia, the structure of anticoagulation management is a pharmacist-managed specialty service. With the current COVID-19 situation, measures were taken to assure the continuity of patient care by establishing tele-pharmacy anticoagulation clinics.

**Materials and Methods:** This was a prospective study with patients prescribed anticoagulation and followed up for 3 months. Since establishing the anticoagulation virtual clinic in March 2020, 270 patients were recruited in the study. The data collected included age, gender, comorbidities, indication for anticoagulation, intended duration of treatment, warfarin dose, testing of International Normalized Ratio (INR), INR target, range of INR values, time INR that was within the therapeutic range (TTR), and complications of therapy (bleeding and/or bruises). The patients were asked to complete the pharmacist satisfaction survey (PSS) after their consultation to assess patient satisfaction with the new virtual consultation system. Linguistic and cultural validation was conducted for the questionnaire.

**Results:** A total of 270 patients were included in the study. The mean percentage of overall INR values in the range was 59.39% ± 32.84, and the mean time with the overall INR was within the therapeutic range 57.81% ± 32.08. Thirty-one percent of the sample had good anticoagulation control (time in therapeutic range >70%). The median satisfaction score was 32 (IQR 28–36) with a maximum score of 40.

**Conclusion:** This is the first study to assess the tele-pharmacy anticoagulation clinic’s efficiency and patient satisfaction in Saudi Arabia during the COVID-19 pandemic. This type of consultation was as effective as face-to-face consultations. The study also highlighted that though the reduction in the cost of care was not substantial, there was a significant increase in resource (clinical pharmacist) utilization as a result of this model. The adoption of tele-pharmacy resulted in time savings for the clinical pharmacists who can be utilized in many other improvement projects in adult ambulatory clinics to ensure the delivery of better quality and safe patient care.

## Introduction

Anticoagulation control for patients taking vitamin K anticoagulants, such as warfarin, is an ongoing concern for patient safety due to the high-risk profile. One method to achieve anticoagulation control is through specialized anticoagulation services. These services are defined as “physicians who manage oral anticoagulation therapy in a systematic and coordinated fashion, incorporating patient education, systematic INR testing, tracking, follow-up, and good patient communication of results and dosing decisions” (Kaatz 2008). In primary care settings and hospital-based anticoagulation clinics, computerized decision-support applications or other means to guide the warfarin dosing may be used. These services provide a systematic and focused approach to improve the quality, safety, and efficacy of delivering anticoagulation care (Bungard et al., 2008). Clinical pharmacist-managed anticoagulation services achieve superior anticoagulation control and have a positive impact. It reduces the rate of hospitalization and emergency department visits due to anticoagulation therapy-related adverse events, resulting in a significant cost-saving in long-term outpatient follow-up (Chiquette et al., 1998; Chamberlain et al., 2001; Rudd and Dier 2010; Manzoor et al., 2017). The most frequently used measure to assess a patient’s anticoagulation control is the International Normalized Ratio (INR) and Time in therapeutic range (TTR) (Reynolds et al., 2004). INR variability measures the stability, and the TTR reflects the achievement of the intensity of anticoagulation. As frequent INRs are obtained when the values are out of range, TTR is the preferred indicator for evaluating anticoagulation quality, as it affects patient outcomes, such as stroke and mortality (Reynolds et al., 2004). In the current study the TTR was calculated *via* the Rosendaal method, and linear interpolation was used to assign an INR value to each day between INR measures over a prolonged interval. Anticoagulation control was defined as good if the mean time of the therapeutic range (TTR) was above 70%, intermediate between 50 and 70%, and inadequate below 50% (Rosendaal et al., 1993).

The current coronavirus pandemic (COVID-19) demanded a new approach to patient care. The use of a tele-medicine model for patient care ensures social distancing and decreases person-to-person contact. It provides convenient access to routine care without the risk of virus transmission (Smith et al., 2020). The World Health Organization defines telemedicine or healing at a distance as “The delivery of health care services, where distance is a critical factor, by all health care professionals using information and communication technologies for the exchange of valid information for diagnosis, treatment and prevention of disease and injuries, research and evaluation, and for the continuing education of health care providers, all in the interests of advancing the health of individuals and their communities” (WHO 2010). The tele-pharmacy model for anticoagulation management can be implemented through the use of telephone-based warfarin dose adjustments based on the INR results in homebound patients. This is an effective method in monitoring high-risk patients with the benefit of decreasing health care expenditure (Hassan et al., 2013). Clinical pharmacists are capable of engaging in and providing comprehensive medication management from a distance (Badowski et al., 2018). Clinical pharmacy services with a tele-pharmacy practice have already been established in many countries, including the United States, Spain, Denmark, Egypt, France, Canada, Italy, Scotland, and Germany (Baldoni et al., 2019).

At King Abdulaziz Medical City (KAMC), Riyadh, Kingdom of Saudi Arabia (KSA), the anticoagulation management structure is a pharmacist-managed specialty service. With the current COVID-19 situation, measures were taken to ensure the continuity of patient care through tele-pharmacy. A coordinator was assigned to the clinic to call all patients 24–72 h before their scheduled visit to remind them to do an INR test before their virtual appointment. Patients were given a choice to do the test in KAMC’s laboratory or outside and send the results to the coordinator *via* WhatsApp. To decrease face-to-face interaction at the anticoagulation clinic visit, the clinical pharmacists interviewed the patient telephonically. They discussed the INR result and the full comprehensive anticoagulation management plan. After the interview, the patients’ virtual visit, including the management plan and future appointment, was documented in the hospital electronic system (BestCare). The prescribed anticoagulant was processed by an ambulatory pharmacist to be sent to patients via the KAMC courier services.

This study assessed the following *1*) tele-pharmacy clinic’s efficiency with respect to mean INR and TTR, *2*) the association between the mean INR and TTR with clinical and demographic variables, *3*) the model’s effectiveness in terms of the rate of bleeding and venous thromboembolism (VTE) episodes, *4*) tele-pharmacy models cost-effectiveness, and *5*) the patient satisfaction with the virtual anticoagulation model.

## Methods

This study was conducted at KAMC, Riyadh, a tertiary care hospital with more than 1,500 beds. It services National Guard employees and their dependents. It provides a wide range of services in all subspecialties, including cardiology, transplant, oncology, and general medicine. Clinical pharmacists manage the anticoagulation clinics and follow more than 700 patients annually. This prospective study was conducted from March 15 to May 30, 2020. As regular clinical data of the patient was taken from the hospital care system, informed consent was waived. Adult patients who were prescribed an anticoagulant for 3 months or more were recruited in the study. These patients were followed for 3 months after the virtual anticoagulation clinic was established in March 2020. The data were collected from the medical records in the hospital Electronic Medical Records System (BestCare). The variables included the age, gender, comorbidities, indication for anticoagulation, intended duration of treatment, warfarin dose, testing of INR (at KAMC or outside), INR target, range of INR values, TTR, and complications of therapy data was collect for 14 months (bleeding or bruises). TTR was calculated via the Rosendaal method, where linear interpolation was used to assign an INR value to each day between INR measures over prolonged interval. Anticoagulation control was defined good if the meantime therapeutic range was above 70%, intermediate if the meantime in therapeutic range was between 50 and 70%, and inadequate if the meantime in therapeutic range was below 50% (Rosendaal et al., 1993).

Patients were asked to complete the “Pharmacist Satisfaction Survey (PSS)” after being evaluated virtually by the clinical pharmacist to assess the patient satisfaction with the new system (Supplementary Material). This questionnaire consists of 10 questions on a 5-point Likert scale from “Not at all” to “very much.” The first seven questions deal with satisfaction with the clinical pharmacist services, and the last three questions with satisfaction with the new virtual clinic system. The survey was adopted from a study that evaluated the impact of a pharmacist-led telemedicine service on chronic disease state management (Maxwell et al., 2016). The primary author granted approval to use the validated survey. The English survey was translated in Arabic and was linguistically validated. Two independent bilingual senior clinical pharmacists translated the original questions from English to Arabic. The two versions were matched, and the differences were adjusted (adverse effects vs. side effects translation, question# 9, medicines vs. treatments). A final version of the Arabic questions was developed and was forwarded to two independent senior clinical pharmacists for “backward” translation. Two independent bilingual senior clinical pharmacists translated the original questions from Arabic to English. The two versions were matched, and the differences were adjusted (question #9). The final versions of the backward translated English and Arabic questions were forwarded to two independent bilingual senior clinical pharmacists for reconciliation (Supplementary Material).

As per the institution directive and precautionary measured applied during the COVID-19 pandemic, all patients were directed to the laboratory services at KAMC for coagulation profile testing before the clinic visit due date except the patient who needed to be seen physically in the clinic (new patients).

Ethics: This study was approved by the Institutional Review Board (IRB) of King Abdullah International Research Center KAIMRC with protocol number RC20/217.

## Analysis

The data was collected in a customized Excel sheet and exported to SPSS. Statistical analysis was performed using the statistical software SPSS v.26 (SPSS Inc., Chicago, IL, United States), and a two-tailed test with *p* < 0.05 was considered statistically significant. The statistical analysis was performed by assessing any missing data for all the variables. The continuous variables were expressed as mean ± standard deviation (SD), and the categorical variables as frequency distributions and percentage. An independent sample *t*-test was used to test the mean TTR and INR with the categorical variables.

The sample was subdivided based on specific characteristics. They were first stratified in age groups, 16–45 yr, 46–65 yr, and >65 yr; gender in male or female, INR: 2–3 and 2.5–3.5; duration of therapy: 0–6 wk and >6 wk; indication for anticoagulation: venous thromboembolism (VTE), including deep venous thrombosis (DVT) and pulmonary embolism (PE), atrial fibrillation (AF) and stroke.

The PSS’s internal consistency (correlation between different items on the same scale or subscale) was assessed using the Cronbach’s alpha coefficient, and a value ≥0.70 was considered acceptable. The convergent and discriminant validity was assessed through factor analysis (Carmines and Zeller 1979; Bhattacherjee 2012). An exploratory factor analysis (EFA) with varimax rotation was performed to examine the latent factor structure (Habing 2003).

## Results

In total, 270 patients were included with a mean age of 58.96 ± 18.43 yr, with 58.9% females. The sample’s demographical and clinical characteristics are displayed in Table 1. Diabetes mellitus (42.6%) and hypertension (49.3%) were the most frequently encountered comorbid conditions. The most prevalent indications for anticoagulation were AF (45.9%), DVT (20.4%), and PE (14.4%). The majority of the sample (94.5%) required lifelong anticoagulation. Warfarin was prescribed in a mean weekly dose of 31.56 ± 17.75 mg. Despite the COVID-19 pandemic and the mandatory curfew, 75.9% of the sample did their coagulation profile testing at KAMC. Most of the patients taking anticoagulation (91.5%) were treated based on a target INR of 2.0–3.0.

**TABLE 1:** Baseline demographic and clinical data of the sample (*n* = 270).

Characteristics	*N* (%)
Age^a^	58.96 ± 18.43 (SD)
**Gender**	
Males	111 (41.1)
Females	159 (58.9)
**Co-morbidities**	
Diabetes	115 (42.6)
Hypertension	133 (49.3)
Hyperlipidemia	56 (20.7)
Ischemic Heart disease	20 (7.4)
Chronic Kidney disease	29 (6.1)
Heart Failure	23 (8.5)
**Indications for Anticoagulation**	
DVT	55 (20.4)
PE	39 (14.4)
AF	124 (45.9)
Stroke	11 (4)
Others	41 (15.3)
**Intended duration of anticoagulation**	
3 mo	3 (1.1)
6 mo	10 (3.7)
12 mo	2 (0.7)
Lifelong	255 (94.5)
**Duration of therapy upon recruitment**	
0–6 wk	10 (3.7)
More than 6 wk	260 (96.3)
Weekly warfarin dose^a^	31.56 ± 17.75
INR test performed	264 (97.8)
**Laboratory for INR testing**	
At KAMC	205 (75.9)
Outside KAMC	65 (24.1)
**Target INR**	
2.0–3.0	247 (91.5)
2.5–3.5	6 (2.2)
Others	17 (6.3)

DVT, deep vein thrombosis; PE, pulmonary embolism; AF, atrial fibrillation.

a Data are given in mean ± standard deviation.

Table 2 describes the anticoagulation control. The mean percentage of the INR values in the therapeutic range was 59.39 ± 32.84%, with the time the INR was within the therapeutic range (TTR) 57.81% ± 32.08. The group who had their first prescription for anticoagulation (0–6 wk) had INR values in the range and a TTR mean of 35.30 ± 23.28% and 38.30% ± 27.83, respectively. The group with AF and the VTE group had lower INR values in the range and a TTR with a mean of 62.65% ± 32.31 and 54.13% ± 30.87, respectively. Regarding age, younger patients (<45 yr) had the lowest INR values in the range and a TTR with a mean of 56.24% ± 34.59 and 52.44% ± 32.76, respectively.

**TABLE 2:** Anticoagulation control.

Clinical Variables	Mean ± SD
**INR values in therapeutic range**	59.39% ± 32.84
**Duration of therapy upon recruitment**	
0–6 wk	35.30% ± 23.28
>6 wk	60.31% ± 32.83
**Indication**	
VTE	54.13% ± 30.87
DVT	55.95% ± 32.32
PE	50.56% ± 29.07
AF	62.65% ± 32.31
Stroke	57.36% ± 39.73
**Age**	
16–45 yr	56.24% ± 34.59
46–65 yr	60.55% ± 30.79
≥65 yr	60.09% ± 34
**Time INR was within therapeutic range(TTR) (Overall)**	57.81% ± 32.08
**Duration of therapy upon recruitment**	
0–6 wk	38.30% ± 27.83
>6 wk	57.98% ± 32.34
**Indication**	
VTE	49.71% ± 29.38
DVT	52.04% ± 31.03
PE	46.21% ± 26.75
A. Fib	62.19% ± 32.29
Ischemic stroke	54.27% ± 37.33
**Age**	
16–45 yr	52.44% ± 32.76
46–65 yr	58.13% ± 30.04
>65 yr	59.27% ± 34.39
**INR Target**	
2–3	59.2 ± 33.02%
2.5–3.5	77.83% ± 17.82

Table 3 describes the proportions of the sample with good to poor anticoagulation control. In total, 31% had a TTR >70%, indicating good control, and 20% had intermediate control. Nearly half of the sample (49.3%) had poor control. Of the sample, 82 had a VTE. In this group, 39.02% had intermediate to good anticoagulation control, and 60.97% poor control. There were 124 patients with AF. In this group, 57.52% had intermediate to good control of anticoagulation. Only 11 patients suffered from a stroke, and 45.45% had good control of anticoagulation.

**TABLE 3:** Percentage of patients with good, intermediate, and poor control.

Clinical Variables	Number (%)
**Overall**	
Time in Therapeutic Range >70%	83 (30.7%)
Time in Therapeutic Range >50% and <70%	54 (20%)
Time in Therapeutic Range <50%	133 (49.3%)
**VTE (per incident)**	82
Time in Therapeutic Range >70%	18 (21.95%)
Time in Therapeutic Range >50% and <70%	14 (17.07%)
Time in Therapeutic Range <50%	50 (60.97%)
**AF**	124
Time in Therapeutic Range >70%	44 (35.48%)
Time in Therapeutic Range >50% and <70%	27 (21.77%)
Time in Therapeutic Range <50%	53 (42.74%)
**Stroke**	11
Time in Therapeutic Range >70%	5 (45.45%)
Time in Therapeutic Range >50% and <70%	3 (27.27%)
Time in Therapeutic Range <50%	3 (27.27%)

Minor bleeding was noticed in 3.67%, and 90.44% did not experience any signs of bleeding. No new VTEs were reported during the 14-month follow-up period for complications (Table 4).

**TABLE 4:** Complication of therapy.

Clinical Variables	Number (%)
**Bleeding**	
Minor	10 (3.67)
Major	8 (2.94)
Bruises	6 (2.20)
None	246 (90.44)

Table 5 describes the *t*-test reported no difference in the mean TTR in groups with and without stroke (TTR 54.76, SD = 37.32% and 57.38, SD = 32.32%, respectively (*p* = 0.76)), and we did not find any difference in the mean TTR between gender and age (age ≤45 and age ≥46 yr). For the groups with and without AF, there was a significant difference in the mean TTR (TTR 62.19%, SD = 32.29% and 53.06, SD-31.92%, respectively (*p* = 0.012)). In addition, there was a difference in the mean TTR in the groups with and without VTE (TTR 49.74%, SD = 29.38 and 60.55%, SD = 33.11%, respectively (*p* = 0.01)).

**TABLE 5:** Significance of mean INR and TTR with respect to variables.

Demographic and clinical variables		INR% (±SD)		TTR% (+SD)
Age				
	16–45	57.78 (34.6)		50.41 (33.84)
	46–65	61.12 (30.35)		29.36 (58.87)
	>65	59.84 (32.6)		56.75 (32.8)
		F = 0.197 (*p* = 0.821)		F = 0.942 (*p* = 0.39)
Gender				
	Male	56.24 (34.59)		55.62 (33.01)
	Female	60.32 (32.32)		58.4 (31.94)
		t = −0.858 (*p* = 0.391)		t = −1.338 (*p* = 0.182)
Afib				
	Yes	63.16 (31.93)		62.19 (32.29)
	No	57.29 (32.99)		53.06 (31.92)
		t = 1.48 (*p* = 0.141)		t = 1.95 (*p* = 0.052)
Ischemic stroke				
	Yes	57.36 (39.72)		54.27 (37.32)
	No	59.47 (32.6)		57.38 (32.2)
		t = −0.208 (*p* = 0.835)		t = −0.312 (*p* = 0.756)
VTE				
	Yes	54.13 (30.86)		49.7 (29.37)
	No	61.68 (33.48)		60.54 (33.1)
		t = −1.742 (*p* = 0.083)		t = −2.558 (*p* = 0.011^a^)
PE				
	Yes	50.56 (29.07)		46.2 (26.75)
	No	61.57 (32.93)		58.72 (32.74
		t = −1.961 (*p* = 0.051)		t = −2.262 (*p* = 0.024^a^)
Duration on recruitment				
	0–6	35.3 (23.28)		38.3 (27.83)
	>6	60.93 (32.55)		57.98 (32.34)
		t = −0.464 (p = 0.01)		t = −1.9 (*p* = 0.06)
INR Target				
	2–3	59.84 (32.75)		57.04 (32.39)
	2.5–3.5	77.83 (17.82)		65.33 (23.76)
		t = −1.338 (*p* = 0.182)		t = −0.623 (*p* = 0.534)

a *p* significant at <0.05.

Table 5 reports a significant difference in the mean INR and the mean TTR between the groups’ demographic and clinical variables. For the groups with and without VTE and the groups with and without PE, the mean TTR was significantly different (*p* < 0.05).

The results of the PSS are presented in Table 6. From the sample (*n* = 270), 265 participants completed the survey without any missing responses. The median satisfaction score was 32 (IQR 28–36) of a maximum score of 40.

**TABLE 6:** Results of survey assessing patient satisfaction with teleconference provided by clinical pharmacists.

Survey Component Assessed	Median Score
Overall median satisfaction score (questions 1–10	32 (IQR 28–36) of a maximum score of 40
Median Pharmacist satisfaction score (questions 1–7)	23 (IQR 20–25) of a maximum score of 28
Median Teleconference satisfaction score (questions 8–10	10 (IQR 7–11) of a maximum score of 12

According to the institution directive and the precautionary measures applied during the COVID-19 pandemic, all the patients were directed to the laboratory services at KAMC for coagulation profile testing before the virtual clinic visit, excluding the group who required a face-to-face consultation (new patients). The redirection of patients to the virtual anticoagulation clinics resulted in negligible cost reduction in coagulation profile at laboratory services. Minimal savings (11%) were noticed in clinical pharmacist costs. A significant reduction (>70%) in the cost of coaguchek (point of care) utilization in the clinic and for nursing services (>80%) was observed. The clinical pharmacist’s cost per patient visit is 200 SAR. One patient is evaluated in an average time of seven (10–15) minutes in usual circumstances. Therefore, 16–24 patients can be seen by one clinical pharmacist in each clinic. In contrast to this, each patient’s time due to tele-pharmacy was reduced to five (5–10) minutes per patient during the COVID-19 pandemic, which resulted in roughly doubling the number of patients (24–48) that could be seen by one clinical pharmacist per clinic.

An EFA was conducted with the 10 items. The three eigenvalues exceeding the unity obtained were 3.91, 1.37, and 1.15. With the three-factor solution, 10 items showed a “simple structure” by having the rotated factors with loadings exceeding 0.5. Five items were loaded on the factor confidence in pharmacist, three items loaded on the experience with telecommunication, and two items loaded on the factor pharmacist advice. The Arabic language PSS had a 3-dimensional factor structure with Confidence, Telecommunication, and Advise from the pharmacist subscales (Table 7 **).**

**TABLE 7:** Pharmacist satisfaction scale: three-factor solution.

	Confidence in Pharmacist	Experience with tele-communication	Pharmacist advise	Item-Total Correlation	Alpha if Item Deleted	Communality
RP aware of Treatment needs	0.859			0.476	0.780	0.742
RP responds to Treatment needs	0.823			0.553	0.779	0.705
RP available to answer questions	0.662			0.546	0.773	0.612
RP help obtain medicine	0.524			0.491	0.778	0.415
Confide in RP	0.521			0.548	0.773	0.513
Lack of physical appearance acceptable		0.869		0.434	0.801	0.765
Comfort on telephone conference		0.847		0.586	0.764	0.767
Encounter is convenient		0.581		0.563	0.768	0.543
RP advises Adverse effects			0.814	0.391	0.788	0.694
RP advices proper use of medicine			0.812	0.382	0.789	0.679

Extraction Method: Principal Component Analysis. Rotation Method: Varimax with Kaiser Normalization.

The internal consistency of the Arabic pharmacist satisfaction scale was good with Cronbach’s alpha coefficients: *α* = 0.78 for the overall PSS total score; *α* = 0.79 for the Confidence subscale (five items); *α* = 0.73 for the telecommunication subscale (six items) and *α* = 0.63 for the Advice subscale (two items). The low Cronbach alpha for the factor Advice of RP is due to only two factor loadings, as this value increases with the number of items within a domain (Taber 2018).

Table 8 describes the convergent and divergent validity. The average variance extracted (AVE) should be >0.5 for the subscales to establish convergent validity. The square root of AVE should be greater than or equal to the correlation estimates (CE) to establish divergent validity between the subscales. Since both these conditions were fulfilled by the PSS instrument, we established the convergent and divergent validity of the instrument.

**TABLE 8:** Convergent and Divergent Validity of scales.

Factors		Factors	Correlation estimate (CE)	Divergent validity (DE ≥ CE	Convergent validity (CE) ≥ 0.5
Confidence in RP	->	Advice of RP	0.421	0.813	0.7
Advice of RP	->	Telecommunication	0.448	0.692	0.5
Telecommunication	->	Confidence in RP	0.253	0.776	0.6

The survey used in our study was reliable, with a Cronbach alpha of 0.8 for all 10 items. The instrument was divided in three factors, each having eigenvalues greater than one. Each factor had minimum loading greater than 0.5, with the highest loading approximately equal to 0.9. We established the divergent validity between the resulting factors, where values for each factor obtained were greater than the correlation estimate between the factors. The convergent validity was also established with values for each factor obtained greater than 0.5.

## Discussion

This study evaluated the tele-pharmacy anticoagulation clinic’s efficiency, cost effectiveness, and the patient satisfaction with the newly established virtual clinic service during the COVID-19 pandemic at KAMC. The mean of the INR values was 60%, and the patients were in the therapeutic range nearly 60% of the time. Of the sample, nearly half achieved intermediate to good anticoagulation control with a TTR above 50%.

A previous retrospective study by Al Yousif et al., conducted at KAMC, Riyadh, with face-to-face consultations with clinical pharmacists in the ambulatory care clinics, reported similar results as the current study (mean INR 62.65 and 31% of patients had TTR>70%) with mean therapeutic INR values of 59% in the patients with AF, and 26% had a TTR >70% (Alyousif and Alsaileek 2016). Similar to the study by Alyousif et al. in the group with and without stroke, the TTR was 56.3 and 60.1%, respectively (*p* = 0.46). The current study did not find a difference in the mean TTR (TTR 54.76 and 57.38 respectively (*p* = 0.76)) and no difference in the mean TTR between gender and age (Table 5) (Alyousif and Alsaileek 2016). In an observational, retrospective study with patients followed up at the Cardiology anticoagulation clinic for AF or VTE, the mean TTR was 60.3% (SD = 19.3%), similar to the current study (57.81% ± 32.08). In the current study, 50% had a TTR>50% compared to 55.7% with a mean TTR >60% in a study by Caldeira et al. (2014). Similar to the study by Caldeira et al., we found the mean INR in the 2.5–3.5 INR target higher than the 2–3 INR target group (77.83 vs. 59.2%). However, there was no significant difference between the groups, which may be due to the small sample size in the 2.5–3.5 INR target group (Table 5). Our results are consistent with several studies evaluating the efficacy and safety of warfarin therapy depending on the percentage of the INR values in the range and the TTR (Chiquette et al., 1998; Wilson et al., 2003; Alyousif and Alsaileek 2016).

We noticed a difference in the proportion of the mean TTR, compared to other studies, because the studies used different cut-off measurements to describe the TTR as excellent, good, and adequate anticoagulation. In the current study, we used the Rosendaal method to describe the percent time in TTR (Rosendaal et al., 1993), which is similar to an observational, retrospective study investigating the efficacy of telephonic dose-adjustments of warfarin in homebound patients. Compared to the current study (mean INR 59.39% and mean TTR = 57.81%), the mean INR in the therapeutic range was 58.39% (SD = 13.96%), and the mean TTR 62.75% (Blissit et al., 2014). Similar to this study, we did not find any difference in the mean INR based on age, gender, or anticoagulation indication, such as AF (Table 5) (Blissit et al., 2014). The results of current study are similar to a study by Macedo et al., where younger age patients (<45 yr) were associated with poor anticoagulation control for AF and VTE patients with a time spent under the INR range of more than 30% (Macedo et al., 2015). In the current study, we found a significant difference in the mean therapeutic INR, based on the duration of the anticoagulation, ≤6 wk or >6 wk, (mean INR 35.30 and 60.93%, respectively, *p* = 0.01). In the current study, for the mean TTR, a significant difference was found between the groups with or without VTE (mean TTR 49.70 and 60.54%, respectively, *p* = 0.01). The current study results, using the tele-pharmacy anticoagulation clinic model, are comparable with literature reporting reasonable control with dedicated anticoagulation clinics with a mean TTR 63% (95% CI 58–68%) (Chiquette et al., 1998; Wilson et al., 2003).

A pooled analysis of 95 AF studies reported that only 56% (95% CI, 53–59%) of the measured INRs were in the therapeutic range, and the patients were 61% (95% CI, 59–62%) of the time in TTR (Mearns et al., 2014). This is comparable to our study, where AF patients had a mean therapeutic INR range of 62.19% ± 32.29 and mean TTR of 62.65 ± 32.31. In real-life clinical practice, only a minority of patients can achieve good anticoagulation control, as demonstrated in a large-scale study population that included 29,717 with AF, in which 44% of the patients had a TTR ≥70% (Macedo et al., 2015). In the current study with 124 AF patients, 35.48% had a TTR >70%. In the current study, with 82 VTE incidents, 22% had a TTR>70%, compared to a study with 19,113 VTE incidents, 36% spent time in TTR >70% (Mearns et al., 2014).

A study reported that when comparing patients already on anticoagulation before the virtual clinic was established, in patients who were newly started on anticoagulation, the mean percentage of the INR values in the therapeutic range was 35 and 61%, respectively, with a TTR of 38 and 58%, respectively. It is expected to have an unstable INR when initiating warfarin for the first few weeks due to highly unpredictable interindividual variability. More frequent testing is optimal to keep patients within the target therapeutic INR, especially during the initial monitoring until the INR response is stable (Horstkotte et al., 1998; Ansell et al., 2008).

During the 14-month follow-up period, there were only 2.94% major bleeding events, and minor bleeding and bruises were reported at a rate of 3.67 and 2.2%, respectively. The vast majority (90.44%) did not develop any adverse events. Although the TTR was 58%, no new VTE cases were reported. A study evaluating the rate of bleeding events in face-to-face versus tele-pharmacy anticoagulation clinics in AF patients reported no significant differences in bleeding events comparing the two types of clinic 6.67 vs. 2.3%, respectively (*p* = 0.304) (Blissit et al., 2014). In a previous cohort conducted in ambulatory care settings, 12% reported significant bleeding, and in 2%, the bleeding was fatal (Landefeld and Goldman 1989), and a second study reported that 52% of the sample had evidence of bleeding (Long et al., 2010). Unfortunately, the previously conducted study at KAMC did not collect bleeding events data to compare the standard care with the newly adopted virtual care in our center (Alyousif and Alsaileek 2016).

A study evaluated the mean TTR for warfarin in patients with AF and VTE. They received pharmacist-managed anticoagulation services and compared patients who were followed-up through face-to-face or telephonic consultations. They did not find significant differences between the two groups, with a mean TTR of 68.17 and 69.57%, respectively (*p* = 0.493) (Blissit et al., 2014). Another study by Staresinic reported a mean percent TTR of 57.8 and 55.1% for face-to-face and telephonic management, respectively (Staresinic et al., 2006). A review reporting the percentage of time in the therapeutic INR range was similar in the face-to-face and in the video clinic models (76.4 versus 80.8%) (Littauer et al., 2017). According to a systematic review of 67 studies that compared the time in the INR range in community practices, anticoagulation clinics and clinical trials reported that the patients were therapeutic 63.6% of the time. This review concluded that monitoring homebound patient’s telephonically was effective or superior to other community-based surveillance modalities (Van Walraven et al., 2006). An Australian study in an inpatient setting demonstrated that tele-pharmacy was as effective as face-to-face medication reviews in identifying problems related to medication in patients at rural inpatient facilities lacking an on-site pharmacist (Littauer et al., 2017).

A study illustrated the financial benefit of homebound monitoring of patients (Hassan et al., 2013). In the current study, the virtual anticoagulation clinic saved 10.5% of the total cost during the 3-month follow-up period, which would result in an annual cost-saving of approximately 80,000 SAR. The resultant time saving can be used in many other improvement projects in adult ambulatory clinics managed by clinical pharmacists. A study illustrated significantly more visits per month for the clinical pharmacy service after implementing the tele-pharmacy anticoagulation clinic. This resulted in the redistribution of workflow, which increased the clinical pharmacy patient volume at the ambulatory care clinics with 16% (Philip et al., 2015; Baines et al., 2018).

The majority of the current studies were satisfied with the tele-pharmacy anticoagulation services. A review reported an adequate level of satisfaction of patients with this model of anticoagulation (Baldoni et al., 2019). A case study evaluated the utilization of clinical video telehealth technology to optimize the clinical pharmacy specialists’ distribution. It illustrated the high-quality anticoagulation management, patient satisfaction, and the optimization of clinical pharmacy specialist resources (Singh et al., 2015). In addition, a study evaluating the impact of pharmacist-led telemedicine on chronic disease state management reported statistically significant diabetes-related therapy goals with very high patient satisfaction (Maxwell et al., 2016).

### Strengths

The clinical pharmacy services delivered with tele-pharmacy offered a quality health care service. We conducted a linguistic and cultural adaptation of the original PSS instrument. This instrument can be used in other anticoagulation care settings to assess patient satisfaction with tele-pharmacy. The prior study, done at the same center with face-to-face anticoagulation clinics, by Yousef et al., allows a comparison with the current data, which supports the effectiveness of the tele-pharmacy anticoagulation clinic model (Alyousif and Alsaileek 2016).

### Limitations

Several factors affected the anticoagulation control in the present study. Nearly half of the patients did not perform their coagulation profile testing 50% of the time during the 3-month follow-up. Secondly, there was a nationwide problem with the local prescription delivery services resulting in a delay, and the patients had no medication, including warfarin, for days. Thirdly, some patients did not respond to the coordinator calls to remind them of the INR test or to the clinical pharmacist call to interview them. Fourthly, the patients treated on a tight INR target of 2–2.5 due to a high risk of bleeding or a target of 2.5–3 due to a high risk of thrombosis were challenging to achieve. Finally, noncompliance was observed with half of the patients who could not achieve a therapeutic INR value. In this study, no analysis was done for individual comorbid conditions as a reason for poor control. In addition, we did not analyze the effect of lifestyle, drug-drug, and drug-food interactions on INR. The patients were only from one tertiary care center located at one location, limiting the generalizability of the results. Other studies indicated a relationship between INR and the number of medications administered (Caldeira et al., 2014). We did not have any information regarding the number and type of medications or the diet. Future studies need to be done with an adequate sample size at multiple sites with a randomized controlled design to establish this model’s effectiveness.

## Conclusion

This is the first study to assess the tele-pharmacy-based anticoagulation clinic’s efficiency, cost reduction, and patient satisfaction in Saudi Arabia during the COVID-19 pandemic. The type of consultation was as effective as face-to-face consultations. We did not observe any significant bleeding or VTE event rates in the sample. The PSS instrument’s cultural and linguistic adaptation was done to measure patient satisfaction with this virtual service. The majority of the patients were satisfied with this model. The study also highlighted that though the financial savings were not considerable, there was a significant increase in resource (clinical pharmacist) utilization. The study illustrated that the services provided by pharmaceutical care could be improved by using a tele-pharmacy model, as this enables the utilization of technology for patients. The adoption of tele-pharmacy resulted in time savings for the clinical pharmacists who can be utilized in many other improvement projects in adult ambulatory clinics to ensure the delivery of better quality and safe patient care.

## Data Availability

The raw data supporting the conclusions of this article will be made available by the authors, without undue reservation.
