# Supplementary material for: Tele-pharmacy Anticoagulation Clinic During COVID-19 Pandemic: Patient Outcomes
Source: Front Pharmacol. 2021 Sep 9;12:652482. doi: 10.3389/fphar.2021.652482 (PMC8459665; doi:10.3389/fphar.2021.652482)
Supplement: Supplementary file 1 [file datasheet2.pdf]

RYD-20-419812-58384

King Abdullah International Medical Research Center
KAIMRC
APPROVED
IRB
Institutional Review Board

Telemedicine-based Anticoagulation Clinic during COVID-19 Pandemic: Patient's outcome

Translated Arabic Survey

Subject ID: ----------------

		أبدا	بعض الأحيان	غالبا	معظم الأحيان	دائما
1	الصيدلي الإكلينيكي يقدم لي النصائح للإستخدام الأمثل للدواء					
2	الصيدلي الإكلينيكي عادة مايشرح لي عن الآثار الجانبية للدواء					
3	لدي ثقة تامة في الصيدلي الإكلينيكي					
4	الصيدلي الإكلينيكي متواجد للإجابة على إستفساراتي					
5	الصيدلي الإكلينيكي يساعدني في ترتيبات الحصول على علاجي					
6	الصيدلي الإكلينيكي المتابع لي على دراية بكل مايتعلق بعلاجي					
7	الصيدلي الإكلينيكي المتابع لي يستجيب لكل مايطرأ على علاجي					
8	مامدى راحتك للتحدث مع الصيدلي من خلال الهاتف					
9	مامدى نقبلك وملائمة طريقة التواصل بالنسبة لك					
10	هل كان عدم التواجد لمقابلة الصيدلي الإكلينيكي بشكل شخصي مقبولا بالنسبة لك					
