# Supplementary material for: Tele-pharmacy Anticoagulation Clinic During COVID-19 Pandemic: Patient Outcomes
Source: Front Pharmacol. 2021 Sep 9;12:652482. doi: 10.3389/fphar.2021.652482 (PMC8459665; doi:10.3389/fphar.2021.652482)
Supplement: Supplementary file 2 [file datasheet3.pdf]

RYD-20-419812-58384

KAIMRC APPROVED IRB
King Abdullah International Medical Research Center
Institutional Review Board

English Version Survey from reference (Pharmacotherapy 2000;20(7):837–843)

Taken with permission

		Not at all	A little bit	Somewhat	Quite a bit	Very much
1	My pharmacist advices me on the proper use of medicines	0	1	2	3	4
2	My pharmacist advices me on the adverse (side) effect of medicine	0	1	2	3	4
3	I have confidence in my pharmacist	0	1	2	3	4
4	My pharmacist is available to answer my questions	0	1	2	3	4
5	My pharmacist helps with the arrangement to obtain my medicine	0	1	2	3	4
6	My pharmacist is aware of my treatment-related needs	0	1	2	3	4
7	My pharmacist respond to treatment-related needs	0	1	2	3	4
8	How comfortable did you feel in talking with someone by video conference	0	1	2	3	4
9	How convenient was the encounter	0	1	2	3	4
10	Was the lack of physical appearance of the provider acceptable	0	1	2	3	4
