# Supplementary material for: Tele-pharmacy Anticoagulation Clinic During COVID-19 Pandemic: Patient Outcomes
Source: Front Pharmacol. 2021 Sep 9;12:652482. doi: 10.3389/fphar.2021.652482 (PMC8459665; doi:10.3389/fphar.2021.652482)
Supplement: Supplementary file 3 [file datasheet1.pdf]

Anticoagulation clinic cost estimation (2019 vs.2020), and COVID-19 pandemic impact

3 months estimation

<u>Coaguocheck cost calculation for 2019:</u>

**Month**	**Average booked and seen in three months**	**20%**	**Cost (x 14)**
Mar-19	343	68.6	960.4
Apr-19	389	77.8	1089.2
Jul-19	414	82.8	1159.2
	**382.00**	**76.4**	1069.6
Month	Average booked walk-in three months	50%	**Cost (x 14)**
Mar-19	161	80.5	1127
Apr-19	197	98.5	1379
Jul-19	144	72	1008
	**167.33**	**83.66666667**	1171.33333
Month	Average walk-in in three months	100%	**Cost (x 14)**
Mar-19	123	123	1722
Apr-19	112	112	1568
Jul-19	177	177	2478
	**137.33**	**137.3333333**	1922.66667
		**Total cost**	4163.6

<u>Coaguocheck cost calculation for 2020:</u>

**Month**	Average booked and seen in three months	20%	Cost (x 14)
Apr-20	0	0	0
Jun-20	54	10.8	151.2
Jul-20	32	6.4	89.6
	28.66666667	5.733333333	80.26666667
Month	Average booked walk-in three months	50%	Cost (x 14)
Apr-20	7	3.5	49
Jun-20	0	0	0
Jul-20	73	36.5	511
	26.67	13.33333333	186.6666667
Month	Average walk-in in three months	100%	Cost (x 14)
Apr-20	56	56	784
Jun-20	69	69	966
Jul-20	52	52	728
	59.00	59	826
		Total cost	1092.933333

Percentage of cost savings in coagucheck = 4164 - 1093= 3071 (74%)

FINAL COST FOR ANTICOAGULATION CLINIC

Average Pt #	2019 (n=686.67)	2020 (n=611)	Difference	COMMENT
Nurse	3433.35 SAR (based on 686.76 CAME)	571.7 SAR (based on 114.34 CAME)	2776.7	Based on the rate of 60SAR/hour (only came)
Clinical pharmacist	137334 SAR	122200 SAR	15134	Based on 200 SAR per patient
Coaguocheck	4164 SAR	1092.933333 SAR	3070.666667	Based on 14 SAR/strip
Pt/INR in the lab	46712 SAR	47651.2 SAR	- 939.2	120 SAR/patient
Total	191643.35 SAR	171515.83 SAR	20127.52 SAR saved	
		% Saved	10.50259245% saved	

Projected cost saving for 12 months:

MONTHS	2019 (COST SAR)	2020 (COST SAR)	Difference (SAVED SAR)	% SAVED
3 months	191643.35	171515.83	20127.52	10.50259245
12 months	766573.4	686063.32	80510.08	42.0103698

Summary:

Virtual anticoagulation clinic implantation had saved 10.5% of the total cost during the study follow up 3 months period. The anticipated cost saving per year will be more than 80,000 SAR (42%). As per the institution directive and precautionary measured applied during COVID-19 pandemic, all patients were directed to laboratory services to do their coagulation profile before the clinic visit due date, unless the patient need to be seen physically in the clinic (new patients), and this had been reflected on a very negligible cost reduction in coagulation profile at laboratory services. Minimal savings (11%) was noticed in clinical pharmacist cost. However, a significant reduction and saving in the cost of more than 70% was noticed in coaguocheck (point of care) utilization in the clinic, and more than 80% in the nurses cost.

The clinical pharmacist cost per patient visit is 200 SAR. At usual circumstances, one patient is usually evaluated in average time of seven (10 -15) minutes, and this had been reflected as 16 -24 patients has been seen by one clinical pharmacist in each clinic. Conversely, this time altered to five (5-10) minutes per patient in virtual clinic workflow during COVID-19 pandemic which resulted in roughly doubling the number of patient (24 – 48) that can be seen by one clinic pharmacist per clinic. This amount of time saving can be utilized in many other improvement projects in adult ambulatory clinics managed by clinical pharmacist.
